# Characterization of Blood Surrogate Immune-Methylation Biomarkers for Immune Cell Infiltration in Chronic Inflammaging Disorders

**DOI:** 10.3389/fgene.2019.01229

**Published:** 2019-11-27

**Authors:** Ken Declerck, Wim Vanden Berghe

**Affiliations:** Laboratory of Protein Chemistry, Proteomics, and Epigenetic Signaling (PPES), Department of Biomedical Sciences, Faculty of Pharmaceutical, Biomedical, and Veterinary Sciences, Antwerp University, Antwerp, Belgium

**Keywords:** deoxyribonucleic acid methylation, inflammaging, atherosclerosis, Alzheimer’s disease, aging

## Abstract

Alzheimer’s disease (AD) and atherosclerosis are both chronic age- and inflammation-dependent diseases. In addition, atherosclerosis is frequently observed in AD patients indicating common involvement of vascular components in both disease etiologies. Recently, epigenome-wide association studies have identified epigenetic alterations, and in particularly DNA methylation changes for both disorders. We hypothesized the existence of a common DNA methylation profile in atherosclerosis and AD which may be valuable as a blood-based DNA methylation inflammaging biomarker. Using publicly available 450k Illumina methylation datasets, we identified a co-methylation network associated with both atherosclerosis and AD in whole blood samples. This methylation profile appeared to indicate shifts in blood immune cell type distribution. Remarkably, similar methylation changes were also detected in disease tissues, including AD brain tissues, atherosclerotic plaques, and tumors and were found to correlate with immune cell infiltration. In addition, this immune-related methylation profile could also be detected in other inflammaging diseases, including Parkinson’s disease and obesity, but not in multiple sclerosis, schizophrenia, and osteoporosis. In conclusion, we identified a blood-based immune-related DNA methylation signature in multiple inflammaging diseases associated with changes in blood immune cell counts and predictive for immune cell infiltration in diseased tissues. In addition to epigenetic clock measurements, this immune-methylation signature may become a valuable blood-based biomarker to prevent chronic inflammatory disease development or monitor lifestyle intervention strategies which promote healthy aging.

## Introduction

Aging and inflammation are important contributors of various chronic lifestyle diseases, including Alzheimer’s disease (AD) and atherosclerosis. Furthermore, AD and atherosclerosis share a lot of disease characteristics and it has been hypothesized that they have a common cause (Lathe et al., 2014).

AD is the most common form of dementia, and is characterized by the accumulation and aggregation of extracellular amyloid-β (Aβ) plaques, the intraneuronal deposition of hyper-phosphorylated tau protein which forms neurofibrillary tangles, neuronal loss, and gliosis in the cerebral cortex and hippocampus (Ballard et al., 2011; Masters et al., 2015). In addition, also vascular components seem to play a crucial role in the initiation and development of AD (Zlokovic, 2011; Nelson et al., 2016; Kisler et al., 2017). The brain consumes a high amount of oxygen and glucose, and therefore the cerebral blood flow is of particular importance for brain health. It is therefore not surprising that cerebrovascular dysfunction has been associated with dementia, AD, and other neurodegenerative disorders. More recently, it has been hypothesized that cerebrovascular damage could be the first hit in AD initiation leading to neuronal injury and loss, and the accumulation of Aβ in the brain, and eventually the development of AD (Zlokovic, 2011).

Atherosclerosis has been associated with dementia and AD (Casserly and Topol, 2004; Lathe et al., 2014). During atherosclerosis development, lipids, macrophages, fibrous connective tissue, and necrotic debris accumulate in the arteries wall leading to the formation of plaques, which can over time rupture and block the blood flow leading eventually to cardiovascular diseases (CVDs) like myocardial infarction or stroke (Lusis, 2000). Both atherosclerosis as AD risk increases with age and has an inflammatory component. Of interest, cerebrovascular atherosclerosis has been found to occur more often in AD patients and correlate with the severity of cognitive impairment (Roher et al., 2003; Roher et al., 2004; Honig et al., 2005; Dolan et al., 2010; Roher et al., 2011; Yarchoan et al., 2012; Yuan et al., 2013; Arvanitakis et al., 2016; Kim et al., 2016). Also carotid atherosclerosis, carotid intima media thickness and coronary artery disease has been associated with AD and AD pathology (Hofman et al., 1997; Beeri et al., 2006; Silvestrini et al., 2009; Silvestrini et al., 2011; Wendell et al., 2012). In addition, adults with CVD show an increased risk for the development of dementia and AD (Newman et al., 2005). Furthermore, atherosclerosis and AD share common risk factors including age, hypertension, type 2 diabetes, obesity, smoking, hypercholesterolemia, and hyperhomocysteinemia (Ballard et al., 2011; Kovacic and Fuster, 2012; Fiolaki et al., 2014). Of interest, in both diseases the APOE4 allele is a genetic risk factor (Yu et al., 2014; Zhu et al., 2016).

Because both diseases are associated with multiple lifestyle and environmental factors, it is not surprising that epigenetic mechanisms are involved in both disease etiologies. Epigenetics is linking environmental factors and genetics through modulation of gene expression patterns. Blood and saliva DNA methylation profiles are increasingly applied as valuable diagnostic and prognostic biomarkers in diseased patients. DNA methylation alterations have been identified in whole blood and plaque tissues of atherosclerosis (Yamada et al., 2014; Zaina, 2014; Zaina et al., 2014; Nazarenko et al., 2015; Valencia-Morales Mdel et al., 2015; Zaina et al., 2015; Istas et al., 2017). Also AD has been associated with methylation changes in blood and different brain regions (De Jager et al., 2014; Lunnon et al., 2014). We recently demonstrated that BRCA1 and CRISPR specific DNA changes in blood can be used as surrogate marker for atherosclerosis (Istas et al., 2017). More particularly, hypermethylation of a CpG island in the promoter region of BRCA1 could be replicated in plaque tissue of two independent cohorts indicating that blood can be used to predict methylation changes in atherosclerotic plaques. Of interest, BRCA1 promoter was also found to be differentially methylated in AD within neurons, and found to be correlated with gene expression (Mano et al., 2017). In AD, however, there is limited evidence that methylation changes in brain tissues are also present in more accessible tissues like blood (Li et al., 2016; Yu et al., 2016). In a study of Lunnon and colleagues, methylation changes found in blood of AD patients were not overlapping with the changes seen in AD brain (Lunnon et al., 2014). However, the AD blood differentially methylated positions (DMPs) were located in the vicinity of genes of relevance to AD and correlated with transcriptional changes making them still potential diagnostic biomarkers.

Given the high commonalities between atherosclerosis and AD disease, here we further examined whether we could find similar DNA methylation signatures in blood of AD and atherosclerosis patients.

## Methods

### Datasets

Genome-wide 450k Illumina DNA methylation datasets were extracted from the Gene Expression Omnibus (GEO) database using the GEOquery R package (Davis and Meltzer, 2007). Raw DNA methylation values were intra-array normalized using the beta mixture quantile dilation (BMIQ) method (Teschendorff et al., 2013) and normalized beta methylation values were used for all further analyses.

**Table 1** summarizes the genome-wide DNA methylation datasets of AD brain and whole blood samples, and plaques and whole blood samples of atherosclerotic patients used in our study. Also, one dataset containing samples of intracranial aneurysm arteries was included. Genome-wide methylation levels were measured using the 450k Illumina arrays in every dataset. The dataset_ID is used to refer to each dataset in the main text.

**Table 1:** Gene Expression Omnibus methylation datasets of Alzheimer’s disease.

Dataset_ID	GEO accession	Disease	Tissue	Sample size
AD_cerebellum_GSE59685	GSE59685	AD	Cerebellum	Controls: 23; cases: 60
AD_EntorhinalCortex_GSE59685	GSE59685	AD	Entorhinal cortex	Controls: 21; cases: 58
AD_FrontalCortex_GSE59685	GSE59685	AD	Frontal cortex	Controls: 24; cases: 60
AD_SupTempGyrus_GSE59685	GSE59685	AD	Superior temporal gyrus	Controls: 26; cases: 61
AD_wholeblood_GSE59685	GSE59685	AD	Whole blood	Controls: 9; cases: 48
AD_cerebellum_GSE72778	GSE72778	AD	Cerebellum	Controls: 9; cases: 23
AD_Frontal_GSE72778	GSE72778	AD	Frontal cortex	Controls: 20; cases: 21
AD_Hippocampus_GSE72778	GSE72778	AD	Hippocampus	Controls: 7; cases: 18
AD_Occipital_GSE72778	GSE72778	AD	Occipital cortex	Controls: 9; cases: 24
AD_TemporalCortex_GSE72778	GSE72778	AD	Temporal cortex	Controls: 6; cases: 23
AD_PrefrontalCortex_GSE80970	GSE80970	AD	Prefrontal cortex	Controls: 68; cases: 74
AD_SupTempGyrus_GSE80970	GSE80970	AD	Superior temporal gyrus	Controls: 70; cases: 74
AD_SupTempGyrus_GSE76105	GSE76105	AD	Superior temporal gyrus	Controls: 34; cases: 34
Athero_wholeblood_GSE107143	GSE107143	Atherosclerosis	Whole blood	Controls: 8; cases: 8
IntracranAneurysm_artery_GSE75434	GSE75434	Intracranial aneurysm	Superficial temporal artery	Controls: 9; cases: 9
AtheroCerebrovas_plaque_GSE66500	GSE66500	Atherosclerosis with cerebrovascular event	Carotid plaque	Controls: 19; cases: 19
Athero_plaquePaired_GSE46394	GSE46394	Atherosclerosis	Aortic plaque	Controls: 15; cases: 15
Athero_plaque_GSE46394	GSE46394	Atherosclerosis	Carotid plaque	Controls: 15; cases: 19

**Table 2** summarizes the genome-wide DNA methylation datasets of whole blood chronic disease samples. Genome-wide methylation levels were measured using the 450k Illumina arrays in every dataset. The dataset_ID is used to refer to each dataset in the main text.

**Table 2:** Gene Expression Omnibus whole blood methylation datasets of different inflammaging diseases.

Accession_ID	Platform	Tissue	Disease	Abbreviation	Sample size
GSE107143	450k	Whole blood	Atherosclerosis	Athero	Controls: 8; cases: 8
GSE59685	450k	Whole blood	Alzheimer’s disease	AD	Controls: 9; cases: 48
GSE72774	450k	Whole blood	Parkinson’s disease	PD	Controls: 219; cases: 289
GSE88824	450k	Whole blood	Multiple sclerosis	MS	Controls: 14; cases: 13
GSE73103	450k	Whole blood	Obesity	Obese	Controls: 268; cases: 87
GSE41169	450k	Whole blood	Schizophrenia	Schizo	Controls: 33; cases: 62
GSE99624	450k	Whole blood	Osteoporosis	Osteo	Controls: 16; cases: 32

### Comparison Alzheimer’s Disease Deoxyribonucleic Acid Methylation Datasets

Genome-wide DNA methylation analysis was performed using the limma moderated t-test (Ritchie et al., 2015) for AD whole blood (GSE59685) and atherosclerosis whole blood (GSE107143) datasets. Genome-wide similarity was determined by correlating the resulting t-statistics using the Pearson’s correlation test.

The DMPs in whole blood samples of atherosclerosis (GSE107143) that we detected previously (Istas et al., 2017) were used to compare with the different AD datasets (whole blood and brain tissues). Athero-DMPs were selected based on an FDR < 0.15 and a delta beta > 0.05 using the limma moderated t-test (Ritchie et al., 2015), resulting in 712 athero-DMPs. Two tailed t-tests were performed to determine the significance level in the AD datasets for each of the 712 athero-DMPs. For each AD dataset, the percentage of overlapping genes (POG) was calculated by dividing the number of athero-DMPs with an unadjusted p-value < 0.05 with the 712 athero-DMPs. The consistency of the overlapping DMPs was calculated by dividing the overlapping DMPs with a same direction of methylation change as in the atherosclerosis whole blood dataset (i.e., hypermethylated in AD dataset and hypermethylated in atherosclerosis dataset) with the total number of overlapping DMPs.

The different DNA methylation datasets were also compared by correlating the t-statistics of each athero-DMP across the datasets using the Pearson’s correlation test.

### Weighted Correlation Network Analysis

To detect co-methylation consensus modules between atherosclerosis and AD in whole blood, the weighted correlation network analysis (WGCNA) R package was used (Langfelder and Horvath, 2008). First, the most variable probes were selected based on an median absolute deviation (MAD) threshold of 0.03 in at least one dataset. In this way 97,375 probes remained for further analysis. The blockwiseConsensusModules function in the WGCNA R package was subsequently used to construct weighted co-methylation networks and detect consensus modules across the two datasets (Zhang and Horvath, 2005). We used the soft-threshold power of 7, a minimum module size of 30 probes, a maximum block size of 20,000, and a dendrogram cut height of 0.25 for module merging as input parameters. The consensus module eigengenes (i.e., first principal component of the module) were associated with disease (either atherosclerosis or AD), and the modules with a significant association (p-value < 0.05) in both datasets were used for further analysis. The module membership of each probe in the modules was calculated by correlating the module eigengenes with the DNA methylation beta values. A module membership close to 1 or −1 indicates high connection with the module. The gene significance values of each probe in the modules were calculated using the t-statistics of the association between the beta-values and the disease groups. The Pearson’s correlation was used to correlate these significance values across different datasets. Probes in the significant modules were mapped to different genomic regions, including gene elements [transcription start site (TSS), gene bodies, untranslated regions (UTRs), intergenic regions] and CpG island (CGI) elements (CGI shelves, shores, and islands) using the Illumina manifest annotation file and Gm12878 ENCODE chromatin segmentation states obtained from the UCSC genome browser. The enrichment of module probes in one of the genomic regions was calculated using the Fisher’s exact test. Probes in the significant modules were mapped to genes using the Illumina manifest annotation file. Pathway enrichment was performed using the Ingenuity Pathway Analysis (IPA) software and the Fisher’s exact test as statistical test.

Module preservation across different AD and CVD datasets were performed using the module Preservation function in the WGCNA R package (Langfelder et al., 2011). One hundred permutations were performed to calculate the preservation z-scores for each dataset. Z-scores higher than 10 indicate strong preservation, between 2 and 10 weak to moderate preservation and below 2 no preservation.

### Estimation of Cell Counts and Immune Cell Infiltration

Cell type fractions were calculated using the method described by Houseman et al. (2012). The EpiDISH R package was used to perform the calculations (Teschendorff et al., 2017). For the whole blood datasets, we used the centDHSbloodDMC.m whole blood reference dataset containing 333 CpG probes of the seven major blood cell types [B-cells, natural killer (NK)-cells, CD4+ T-cells, CD8+ T-cells, granulocytes, and monocytes]. To estimate the cell counts in the atherosclerosis vascular tissues, we created a new reference methylome. For the smooth muscle cells, fibroblasts, and endothelial cells, we retrieved 450k Illumina methylation data of AoSMC, ProgFib, and HUVEC from the ENCODE project (GSE40699), respectively. Raw methylation values were intra-array normalized using the BMIQ method (Teschendorff et al., 2013) and normalized beta-values were subsequently used for further analysis. Immune cell (IC) reference methylomes were obtained from the study of Reinius et al. (2012). Next, differences in methylation across the different cell types were calculated using limma linear models (Ritchie et al., 2015) comparing each cell type with the rest of the samples: IC *vs.* rest of samples, AoSMC *vs.* rest of samples, ProfFib *vs.* rest of samples, and HUVEC *vs.* rest of samples. For each cell type the top 100 significant CpG probes with the largest methylation difference were selected and combined to obtain 357 unique CpG probes. The beta values of the ICs were averaged to obtain the final reference methylome (**Supplementary Table 1**). This reference methylome was subsequently used to estimate cell counts and IC infiltration in the vascular tissues using the reference based method described by Houseman et al. (2012) and implemented in the EpiDISH R package (Teschendorff et al., 2017). Information about IC infiltration of The Cancer Genome Atlas (TCGA) cancers were obtained from a recent study examining immunogenomic profiles of different cancers (Thorsson et al., 2018). TCGA level-3 450k Illumina methylation data were retrieved using the TCGAbiolinks R package (**Table 3**) (Colaprico et al., 2016). Beta-values were used for subsequent analyses.

**Table 3:** The Cancer Genome Atlas datasets.

TCGA code	TCGA name	Sample size
ACC	Adrenocortical carcinoma	Controls: 0; cases: 80
BLCA	Bladder urothelial carcinoma	Controls: 21; cases: 419
BRCA	Breast invasive carcinoma	Controls: 96; cases: 796
CESC	Cervical squamous cell carcinoma	Controls: 3; cases: 309
CHOL	Cholangiocarcinoma	Controls: 9; cases: 36
COAD	Colon adenocarcinoma	Controls: 38; cases: 315
DLBC	Lymphoid neoplasm diffuse large B-cell lymphoma	Controls: 0; cases: 48
ESCA	Esophageal carcinoma	Controls: 16; cases: 186
GBM	Glioblastoma multiforme	Controls: 2; cases: 153
HNSC	Head and neck squamous cell carcinoma	Controls: 50; cases 530
KICH	Kidney chromophobe	Controls: 0; cases: 66
KIRC	Kidney renal clear cell carcinoma	Controls: 160; cases: 325
KIRP	Kidney renal papillary cell carcinoma	Controls: 45; cases: 276
LAML	Acute myeloid leukemia	Controls: 0; cases: 140
LGG	Brain lower grade glioma	Controls: 0; cases: 534
LIHC	Liver hepatocellular carcinoma	Controls: 50; cases: 380
LUAD	Lung adenocarcinoma	Controls: 32; cases: 475
LUSC	Lung squamous cell carcinoma	Controls: 42; cases: 370
MESO	Mesothelioma	Controls: 0; cases: 87
OV	Ovarian serous cystadenocarcinoma	Controls: 0; cases: 10
PAAD	Pancreatic adenocarcinoma	Controls: 10; cases: 185
PCPG	Pheochromocytoma and paraganglioma	Controls: 3; cases: 184
PRAD	Prostate adenocarcinoma	Controls: 50; cases: 503
READ	Rectum adenocarcinoma	Controls: 7; cases: 99
SARC	Sarcoma	Controls: 4: cases: 265
SKCM	Skin cutaneous melanoma	Controls: 2; cases: 473
STAD	Stomach adenocarcinoma	Controls: 2: cases: 395
TGCT	Testicular germ cell tumors	Controls: 0; cases: 156
THCA	Thyroid carcinoma	Controls: 56; cases: 515
THYM	Thymoma	Controls: 2: cases: 124
UCEC	Uterine corpus endometrial carcinoma	Controls: 46; cases: 439
UCS	Uterine carcinoma	Controls: 0; cases: 57
UVM	Uveal melanoma	Controls: 0; cases: 80

## Results

### Atherosclerosis and Alzheimer’s Disease Whole Blood Samples Contain a Common Deoxyribonucleic Acid Methylation Signature

**Figure 1:**
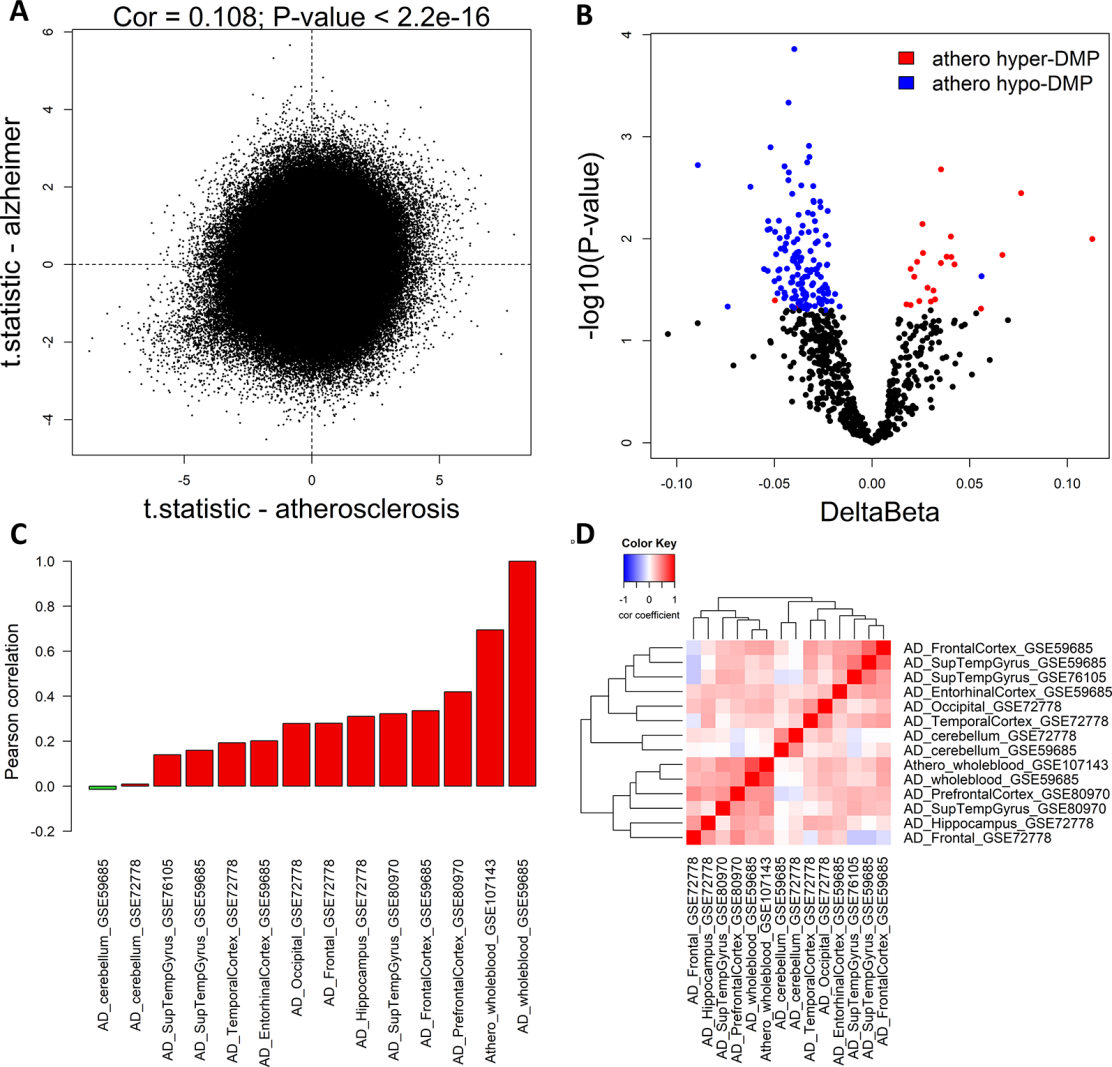
Common DNA methylation signature in atherosclerosis and Alzheimer’s disease (AD) whole blood and brain samples. **(A)** The genome-wide significance levels (t-statistic) of each CpG-probe in the atherosclerosis (GSE107143) and AD (GSE59685) whole blood dataset was plotted in the X-axis and Y-axis, respectively. The Pearson correlation was used to calculate the correlation between the two datasets. **(B)** Volcano plot showing the methylation differences and statistical significance values after comparing the methylation values of the 712 top significant atherosclerosis differentially methylated positions (DMPs) (athero-DMPs) between AD patients and healthy individuals. Probes which were significantly different between AD and controls (p-value < 0.05) were colored blue when hypomethylated and red when hypermethylated in atherosclerosis. **(C)** Correlation coefficients between the t-statistics of the 712 athero-DMPs in the atherosclerosis whole blood dataset and the t-statistics of the athero-DMPs in multiple AD brain and whole blood datasets. Positive correlations are represented as a red bar, and negative correlations as a green bar. **(D)** Correlation heatmap representing the correlation coefficients between the t-statistics of the 712 athero-DMPs across different AD datasets. Red means a positive correlation and blue a negative correlation.

To compare methylation profiles between atherosclerosis (GSE107143) and AD (GSE59685) in whole blood, we first compared the genome-wide significance of each CpG probe in both datasets. Using the limma moderated t-test, we performed differentially methylation analysis on both whole blood datasets. We found a weak positive correlation between the t-statistics (Pearson correlation: 0.108, P < 2.2e^−16^) in both datasets, indicating that at the genome-wide level the similarity between the methylation profiles in atherosclerosis and AD is limited (**Figure 1A**). Next, we checked more specifically, whether the top significant CpG-probes found in our atherosclerosis dataset (Istas et al., 2017) were also differentially methylated in the AD dataset. We first selected the most significant CpG-probes by setting the threshold for differentially methylation at FDR < 0.15 and delta beta > 0.05. In this way 712 CpG-probes were selected which we called athero-DMPs. T-tests were performed to determine the significance level in the AD dataset for each of the 712 athero-DMPs. We found several probes which were also found to be differentially methylated in AD (p-value < 0.05) (**Figure 1B**). Of particular interest, the directionality of the methylation change was very similar in both datasets.

Using different publicly available DNA methylation datasets (**Table 1**), we performed the same analyses in multiple AD brain tissues. For each AD dataset, we selected the 712 athero-DMPs and correlated the t-statistics resulted from the comparison in methylation between AD patients and healthy controls. Again, for some of the brain tissue we could find a similar methylation profile compared to atherosclerosis blood samples (**Figures 1C, D** and **Supplementary Figure 1**). Especially in frontal lobe, frontal cortex, and superior temporal gyrus, the hypo- and hypermethylated atherosclerosis DMPs corresponded with hypo- and hypermethylation in AD, respectively (**Supplementary Figure 1**). The POG in each tissue was rather limited, with values ranging between 4 and 32% (**Table 4**). However, the direction of methylation change for most of the overlapping CpG sites were highly consistent with the atherosclerosis whole blood dataset (consistency %) (**Table 4**). For example, 23.2% of athero-DMPs were also found significant in AD whole blood samples of which 98.2% of the DMPs were directional consistent with the athero-DMPs. The percentage of consistency was the lowest for the AD cerebellum samples (49.4 and 61.8%) and a dataset with AD superior temporal gyrus samples (45.1%). Except for cerebellum tissues, there was a positive correlation between the t-statistics of the athero-DMPs in the atherosclerosis whole blood dataset and the other AD brain and whole blood datasets, with correlation coefficients ranging from 0.14 to 0.69 (**Figure 1C**). In contrast, in cerebellum samples no strong correlation could be found (correlation coefficient ∼ 0). Furthermore, cerebellum samples did not correlate with the other brain AD tissues (**Figure 1D**).

**Table 4:** Percentage of overlapping genes (POG) and consistency.

Dataset_ID	POG %	Consistency %
AD_Frontal_GSE72778	31.9	81.5
AD_wholeblood_GSE59685	23.2	98.2
AD_SupTempGyrus_GSE76105	16.0	45.1
AD_SupTempGyrus_GSE59685	13.9	76.8
AD_FrontalCortex_GSE59685	12.9	84.8
AD_cerebellum_GSE72778	12.5	61.8
AD_cerebellum_GSE59685	11.7	49.4
AD_SupTempGyrus_GSE80970	10.3	82.2
AD_Hippocampus_GSE72778	10.1	69.4
AD_PrefrontalCortex_GSE80970	9.8	78.6
AD_TemporalCortex_GSE72778	9.7	75.4
AD_EntorhinalCortex_GSE59685	5.3	84.2
AD_Occipital_GSE72778	3.9	78.6

### An Atherosclerosis-Alzheimer’s Disease Whole Blood co-Methylation Consensus Network Is Enriched for T-Cell Regulatory Pathways

We next used WGCNA to identify a consensus co-methylation module of atherosclerosis and AD in whole blood. One hundred seventy-five consensus modules could be found. We used the module eigengenes to associate the different modules with atherosclerosis and AD disease state. We found 25 and 16 modules significantly associated with atherosclerosis and AD, respectively. Three consensus modules were found to be both significant (p-value < 0.05) in atherosclerosis and AD. Module ME91 was positively associated with atherosclerosis and negatively with AD, module ME54 was positively associated in both datasets, and module ME21 was negatively associated in both datasets.

**Figure 2:**
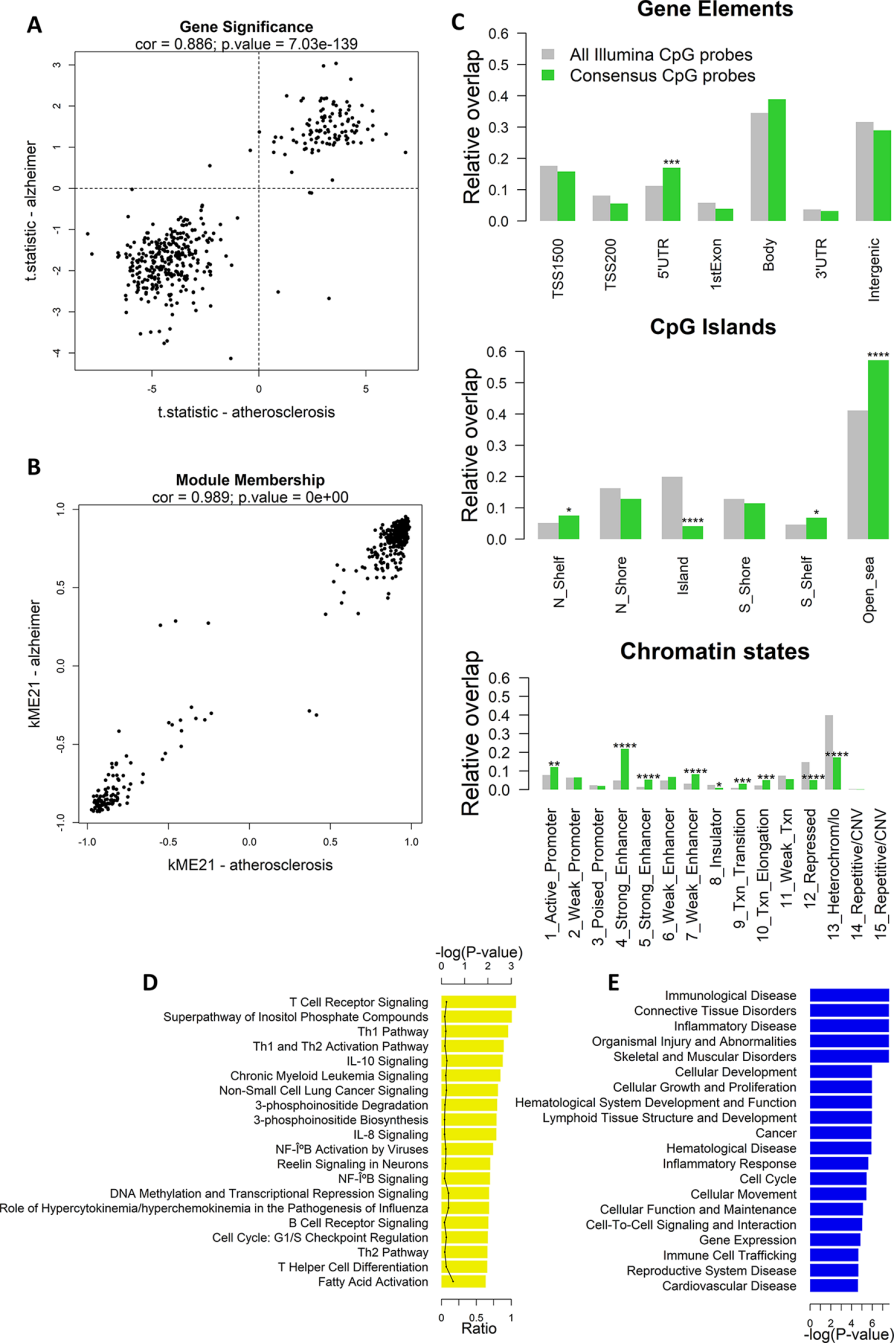
Weighted Correlation Network Analysis (WGCNA) co-methylation consensus module in atherosclerosis and Alzheimer’s disease (AD) whole blood datasets. **(A)** Correlation of gene significance values (t-statistics) of CpG probes in the consensus module (module ME21) between atherosclerosis and AD whole blood datasets. The Pearson’s correlation coefficient and p-value are provided at the top of the scatterplot. **(B)** Correlation of module membership of CpG probes in the consensus module (module ME21) between atherosclerosis and AD whole blood datasets. The Pearson’s correlation coefficient and p-value are provided at the top of the scatterplot. **(C)** Genomic enrichment of the consensus module CpG probes (module ME21) in multiple genomic regions: gene elements (top), CpG island elements (center), and chromatin segmentation states (bottom). CpG probes in module ME21 were mapped to different genomic regions and enrichment or depletion compared to all Illumina CpG probes was determined using the Fisher’s exact test. Green bars represent the relative overlap of consensus module ME21 CpG probes with the genomic regions, while gray bars represent the relative overlap of all Illumina CpG probes with the genomic regions. * Fisher’s exact P ≤ 0.05, ** P ≤ 0.01, *** P ≤ 0.001, **** P ≤ 0.0001. **(D)** Significantly enriched Ingenuity Pathway Analysis (IPA) canonical pathways, and **(E)** IPA diseases and biofunctions of genes containing a consensus module CpG probe. Enrichment was calculated using the Fisher’s exact test.

Next, we calculated the gene significance and module membership of the probes in the consensus modules. We defined the gene significance as the t-statistic of the association between the CpG-probe methylation value and disease state, and the module membership as the correlation coefficient between the module eigengene and the CpG-probe methylation beta value. The closer the module membership is to 1 or −1 the more important the probe is in the module. In general, a module membership close to 1 or −1 is highly connective and therefore represents a hub in the network. There was a strong correlation between the gene significance in the two datasets for module ME21 (Pearson’s correlation: 0.845) (**Figure 2A**). The same was true for the module membership (Pearson’s correlation: 0.989) (**Figure 2B**). As expected the gene significance and module membership was also highly correlated (**Supplementary Figure 2**). Because a less strong correlation could be found with module ME54 (data not shown), we decided to focus only on module ME21 as the consensus module (**Supplementary Table 2**).

We next mapped the CpG probes in the consensus module to different genomic regions relative to gene elements (TSS, gene bodies, etc.), CGIs, and chromatin segmentation states. Interestingly, we found an enrichment in 5’UTR regions, CpG-poor regions outside CGIs, active promoters, strong and weak enhancers, transcriptional transition, and elongation states (**Figure 2C**). In addition, the consensus module CpG probes were strongly depleted in CGIs, repressed chromatin states, and heterochromatin.

The consensus module CpG probes were subsequently mapped to genes. IPA pathway analysis showed a strong enrichment in T cell regulatory and immune pathways, including T- and B cell receptor signaling, Th1 and Th2 pathway, IL-10 and IL-8 signaling, and NF-κB signaling (**Figure 2D**). In addition, genes were enriched in immunological and inflammatory diseases, and functions related to cellular development, growth, proliferation, and movement (**Figure 2E**).

### The Atherosclerosis-Alzheimer’s Disease Blood Consensus Network Is Also Associated in Brain Tissues and Atherosclerotic Plaques

**Figure 3:**
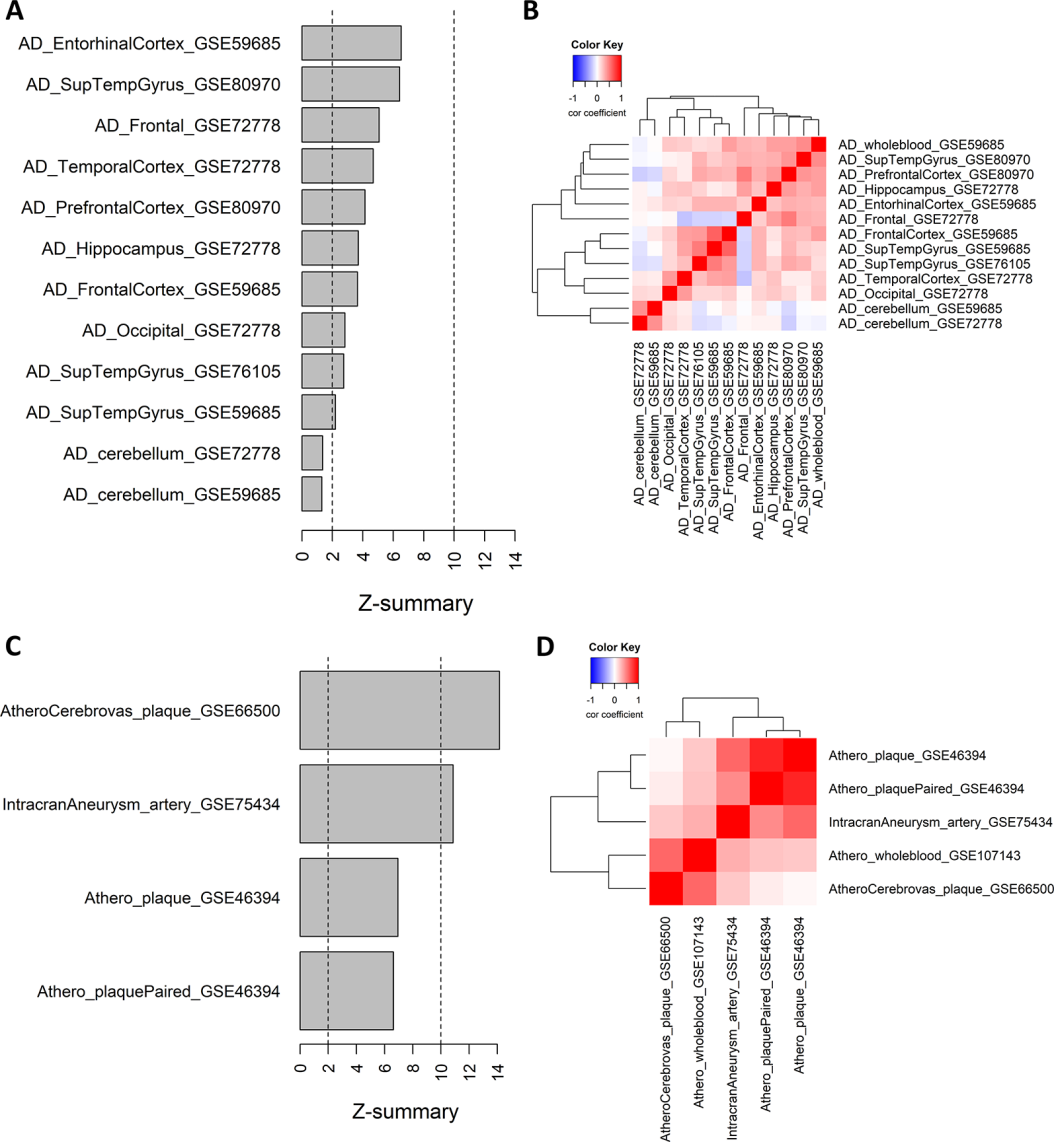
Weighted Correlation Network Analysis consensus module in Alzheimer’s disease (AD) brain tissues and atherosclerotic plaques. **(A)** Consensus module ME21 preservation in AD tissue datasets. For each dataset the preservation z-score is shown. Z-scores below 2 indicates no preservation, between 2 and 10 weak to moderate preservation and above 10 strong preservation. Info about the datasets can be found in **Table 1**. **(B)** Correlation heatmap representing the Pearson’s correlation coefficients between the gene significance values (t-statistics) of the consensus module ME21 CpG sites across different AD datasets. Red means a positive correlation and blue a negative correlation. **(C)** Consensus module ME21 preservation in atherosclerosis and cardiovascular disease (CVD) datasets (see **Table 1**). **(D)** Correlation heatmap representing the Pearson’s correlation coefficients between the gene significance values (t-statistics) of the consensus module CpG sites across different CVD datasets. Red means a positive correlation and blue a negative correlation.

We further analyzed whether module 21 was preserved in other AD methylation datasets of different brain tissues. The preservation z-scores for all AD brain tissues, except for cerebellum, were between 2 and 10, suggesting weak to moderate preservation (**Figure 3A**). In cerebellum, there was no indication of module preservation (z-score < 2). We next calculated for each AD dataset the gene significance values (t-statistics) of the CpG probes in the consensus module, and performed pairwise correlation across the different AD datasets. Except for cerebellum, all the other AD datasets showed a positive correlation with the gene significance values of the whole blood datasets, and relative to each other (**Figure 3B**).

We next wondered whether the same pattern could also be found in other methylation datasets related to CVD and atherosclerosis. We extracted 450k Illumina data from carotid plaques, plaques after cerebrovascular event and arteries with intracranial aneurysm (**Table 1**). Here the preservation was much stronger, with z-scores higher than 10 in the atherosclerotic plaques after a cerebrovascular event and in intracranial aneurysm, while for the carotid plaque datasets we found moderate module preservation (**Figure 3C**). Again, we could find strong positive correlations between the gene significance values of the different datasets (**Figure 3D**). Of note, the highest correlation with the whole blood dataset could be found with the atherosclerotic plaque dataset with a cerebrovascular event. In contrast, there was no evidence of correlation between the carotid plaque dataset and the dataset with a cerebrovascular event.

### The Atherosclerosis-Alzheimer’s Disease Blood Consensus Network Represents a Common Immuno-Methylation Signature

**Figure 4:**
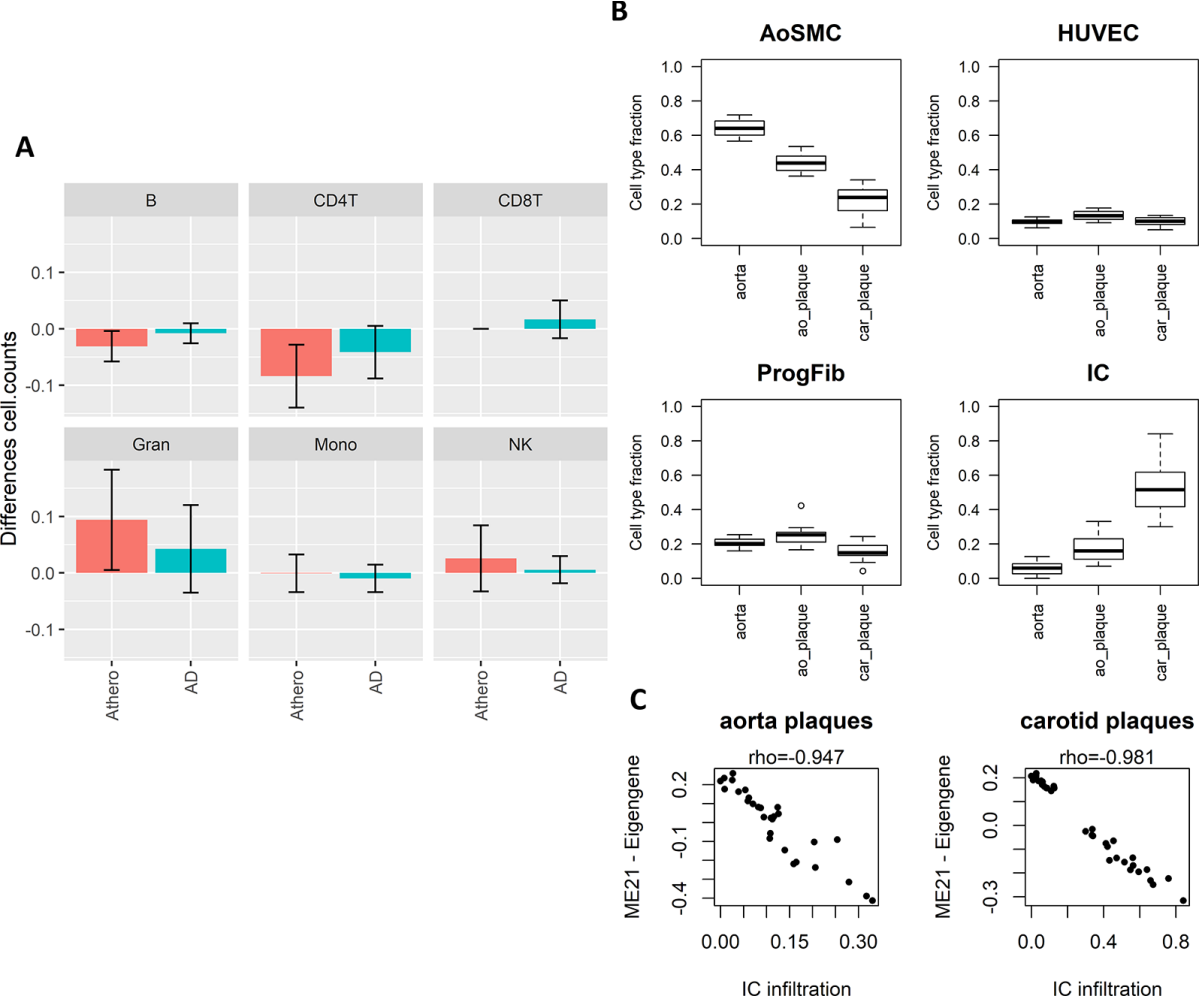
Weighted Correlation Network Analysis (WGCNA) consensus module is influenced by underlying immune cell type composition and immune cell infiltration. **(A)** Blood immune cell type (B-cells, NK-cells, CD4+ T-cells, CD8+ T-cells, granulocytes, and monocytes) composition shift in atherosclerosis (blue) and Alzheimer’s disease (AD) (green) patients compared to controls estimated by the method of Houseman. Error bars represent 95% confidence intervals. **(B)** Estimated cell type composition in healthy aorta, aorta atherosclerotic plaque (ao_plaque), and carotid plaque (car_plaque). Relative cell type composition was estimated using reference methylomes of aortic smooth muscle cells (AoSMC), endothelial cells (HUVEC), fibroblasts (ProgFib), and immune cells (IC) (see *Methods* for more details). **(C)** Correlation between estimated IC infiltration and the eigengenes of the WGCNA consensus module (ME21) in aorta and carotid plaques. A negative correlation was found between estimated IC infiltration and ME21 eigengenes, which corresponds with the negative association found between module ME21 eigengenes and methylation in AD and atherosclerosis (i.e., AD and atherosclerosis patients have lower ME21 eigengenes compared to controls).

The enriched pathways in T cell activation and function indicate that part of the methylation changes may be due to differences in cell type heterogeneity in the samples analyzed. We therefore estimated cell type composition in the atherosclerosis and AD whole blood datasets. B-cells and CD4+ T-cell levels were both reduced while granulocyte levels were increased in atherosclerosis and AD samples as compared to healthy blood samples (**Figure 4A**). In addition, many CpG probes in the consensus module seem variable across the different blood IC types (**Supplementary Figure 3**). In this respect, the positive correlations seen with the AD brain and atherosclerotic plaque tissues could reflect the infiltration of IC in the brain and arterial wall respectively. To verify this hypothesis, we estimated IC fraction in the vascular tissues using a new reference methylome created from methylation profiles of aortic smooth muscle cells (AoSMC), fibroblasts (ProgFib), human umbilical vein endothelials cells (HUVEC), and IC (see Methods section for details). As expected, an increase in IC was observed in plaque tissue compared to healthy aorta tissue (**Figure 4B**), and monocytes were the main infiltrated blood cell type observed in the plaques (**Supplementary Figure 4**). More surprisingly, the AoSMC fraction was relatively lowered in plaque material. HUVEC and ProgFib fractions didn’t show substantial differences. Methylation values from the reference methylomes of the 500 most significantly DMPs in aorta plaques revealed that the hypermethylated profile in atherosclerotic plaques (Zaina et al., 2014) was mainly due to an overall hypermethylation in IC compared to the other cell types. Similarly, the small fraction of hypo-DMPs could also be attributed to hypomethylated CpG sites in IC (**Supplementary Figure 5**). Furthermore, a strong correlation was found between the consensus module eigengene and the estimated IC fraction in aorta and carotid atherosclerotic plaque tissues, supporting our hypothesis (**Figure 4C**).

To further prove that our methylation profile measures an immune component, we made use of IC infiltration information of TCGA cancers obtained from a recent study (Thorsson et al., 2018). As expected, in almost all cancers there was a negative correlation between the module eigengenes and leukocyte fraction, stromal fraction, and lymphocyte infiltration signature score (**Supplementary Figure 6**). Thus, tumors with methylation profiles resembling the methylation consensus module demonstrated more IC infiltration. This is completely in line with our observations and supports our conclusions.

### The Consensus Methylation Module Is Also Present in Other Chronic Inflammaging Diseases

**Figure 5:**
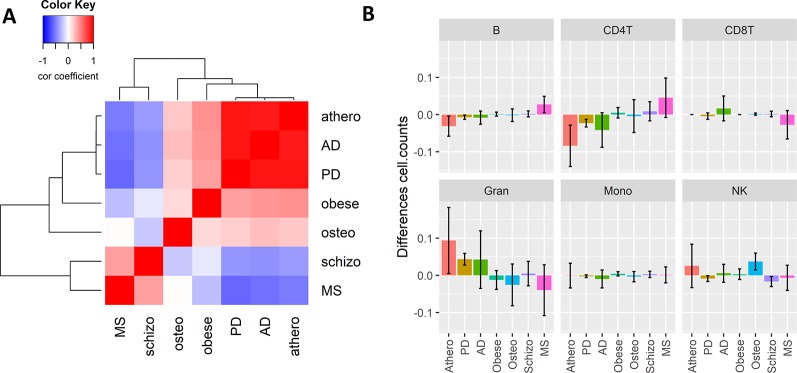
The Weighted Correlation Network Analysis co-methylation consensus module in whole blood of other inflammaging diseases. **(A)** Correlation heatmap representing the Pearson’s correlation coefficients between the gene significance values (t-statistics) of the consensus module ME21 CpG sites across six chronic inflammaging diseases: atherosclerosis (athero), Alzheimer’s disease, Parkinson’s disease, obesity (obese), osteoporosis (osteo), schizophrenia (schizo), and multiple sclerosis (see **Table 2**). Red means a positive correlation and blue a negative correlation. **(B)** Estimated blood immune cell type composition shifts in the different chronic inflammaging diseases compared to healthy controls. Error bars represent 95% confidence intervals.

Since inflammation is a common hallmark of many chronic aging diseases, we further checked whether the consensus immuno-methylation module identified was also prevalent in other chronic inflammation and age-dependent diseases. We therefore reanalyzed Illumina 450K DNA methylation profiles of whole blood samples of Parkinson’s disease (PD), schizophrenia, obesity, osteoporosis, and multiple sclerosis (MS) (**Table 2**), and correlated the gene significance values of the CpG probes in the consensus module across all the diseases (**Figure 5A**). A strong positive correlation could be observed with PD (r: 0.91). Also obesity (r: 0.43) and osteoporosis (r: 0.21) showed a moderate positive correlation. On the other hand, schizophrenia (r: −0.39) and MS (r: −0.51) demonstrated a negative correlation.

The high correlation found in PD could again be attributed to a shift in CD4+ T cell and granulocyte blood levels. However, no such changes were detected in obesity and osteoporosis (**Figure 5B**). In osteoporosis, NK cell levels were slightly higher as compared to healthy samples. In obesity and schizophrenia, IC types didn’t change dramatically. MS patients had opposite cell type distribution in comparison to atherosclerosis, AD and PD patients with higher CD4+ T- and B-cell levels and lower granulocyte levels.

## Discussion

In this study, we identified a common DNA methylation signature in whole blood of atherosclerosis and AD patients. We showed that this consensus methylation module represents an immune component which correlates with shifts in blood IC distribution and IC infiltration in plaques and brains. Finally we demonstrate the applicability of the immune-methylation signature, as an inflammaging disease biomarker. This study provides evidence that IC type counts measured by DNA methylation may be a useful way to monitor age- and inflammation related diseases such as AD and CVDs. In this regard, DNA methylation may be a very sensitive method of measuring the immune status of a tissue and detecting subtle changes in cell type composition and cellular activation states.

Blood-based DNA methylation biomarkers can be valuable for diagnostic, predictive, prognostic, and therapeutic purposes (Horvath and Raj, 2018; Berdasco and Esteller, 2019). Here, we showed that blood DNA methylation in atherosclerosis and AD are associated with similar shifts in IC type distribution and/or tissue infiltration. In both atherosclerosis and AD, granulocyte levels were increased while B and CD4+ T-cells were decreased. This is in accordance with other studies showing a higher neutrophil/lymphocyte ratio (NLR) in these diseases (Kuyumcu et al., 2012; Balta et al., 2016). NLR is a marker of systemic inflammation and has been found to be prognostic marker in CVDs associated with poor outcome and mortality (Teperman et al., 2017; Xue et al., 2017). Interestingly, NLR can also be used to predict the presence of carotid atherosclerotic plaques (Corriere et al., 2018). Also in AD, NLR was higher as compared to healthy controls (Kuyumcu et al., 2012). However, strong evidence for NLR as a prognostic or predictive biomarker in AD is lacking (Rembach et al., 2014). Whether our methylation profile is also a predictor of poor outcome or disease severity should be further investigated.

In cancer, systemic inflammation is associated with poor outcome (Diakos et al., 2014; Rossi et al., 2017). A recent study used DNA methylation to estimate NLR (Koestler et al., 2017), and found that this methylation-derived NLR (mdNLR) was associated with poor survival in various cancer types (Koestler et al., 2017; Wiencke et al., 2017). Furthermore, they also showed that mdNLR was increased with age (Koestler et al., 2017). Indeed, age is also accompanied by chronic low-level systemic inflammation, which is often called inflammaging (Franceschi et al., 2018b). In addition, many chronic diseases are more common with higher age, and it has therefore been suggested that aging and age-associated chronic diseases share the same underlying biological mechanisms (Kennedy et al., 2014; Franceschi et al., 2018a). Many age-associated chronic diseases can therefore been seen as an acceleration of the aging process. Epigenetic clock age can be deduced from Illumina 450K DNA Methylation profiles and accelerated epigenetic clock age has been associated with mortality and age-related diseases and phenotypes, suggesting that the epigenetic clock is a measure for biological age, rather than chronological age (Declerck and Vanden Berghe, 2018; Horvath and Raj, 2018). Interestingly, no single CpG site was in common between the immune-methylation signature identified in this study and the epigenetic clock signature, indicating a difference between the two DNA methylation-based biomarkers. Therefore, we also tested whether our methylation profile was present in other inflammation- and aging-associated diseases, besides AD and atherosclerosis. Remarkably, we observed a similar immunomethylation related change in cell type contribution. In contrast, the other diseases tested showed either low association with our methylation profile or no association, indicating that this profile is not a general marker for all inflammaging diseases. For example, MS showed a rather negative correlation with our methylation profile, which was also reflected in an opposite shift of cell type distribution, with higher lymphocytes and lower granulocytes levels. In contrast to MS, obesity showed a mild positive correlation, although this does not change the cell type contribution of the major blood cell types, which may indicate the involvement of other minority blood cell types or different activation cell activation states (Defuria et al., 2013; Touch et al., 2017). Indeed, we only used the major IC types extracted from the study of Reinius, and it can be anticipated that with the generation of more reference methylomes of major and minor blood cell types a more complete picture of subtle cell type effects can be detected. In addition, other techniques beside the well-known houseman approach to estimate cell type composition, such as CIBERSORT (Newman et al., 2015) often used in gene expression studies and robust partial correlations (RPC) may improve cell type composition estimation (Teschendorff et al., 2017).

In most blood-based epigenome-wide association studies (EWAS), the Houseman algorithm is frequently applied to correct for variations in blood sample cell composition which may contribute to methylation variability (Jaffe and Irizarry, 2014). However, we believe that this immune component may be an important determinant of aging disease etiologies and holds valuable information for prognostic or therapeutic biomarker applications. DNA methylation may be a very sensitive method to estimate small shifts in IC distribution or activation status. For example, a recent study found DNA methylation differences were associated with NK cell activation (Wiencke et al., 2016). DNA methyltransferase DNMT3B seems to be important in regulating macrophage polarization (Yang et al., 2014). In another study, FOXP3 methylation can be used to count regulatory T cells in blood and solid tissues (Wieczorek et al., 2009). A methylation CpG site in GPR15 gene which was associated with smoking, was found to be due to a higher proportion of CD3+GPR15+ expressing T cells in blood, and not by the direct effect of smoking on DNA methylation (Bauer et al., 2015). Correcting for cell type effects in EWAS is not always useful and may remove important information about the disease pathology (Holbrook et al., 2017). In addition, even highly purified cell types were found to be rather a collection of epigenomes (which the authors called meta-epigenomes) (Wijetunga et al., 2014), and may therefore not exclude all cellular effects. The usefulness of measuring cell type effects using DNA methylation was also exemplified by the extrinsic epigenetic clock which is influenced by blood cell counts. Faster extrinsic epigenetic age acceleration was associated with all-cause mortality (Chen et al., 2016), while different healthy lifestyle factors resulted in a decrease in extrinsic epigenetic age acceleration (Quach et al., 2017). These results indicate that it may be useful to also include cellular effects which may be used to asses therapeutic, nutritional, and lifestyle interventions. Therefore, removing cell type effects in EWAS is not always preferable and may ignore important contributors of chronic diseases, as can be seen in this study.

Although this immune-associated DNA methylation profile is associated with atherosclerosis and AD, further longitudinal studies are required to estimate whether it is also related to disease outcome or progression. We established a correlation of DNA methylation changes with IC infiltration in atherosclerotic plaques and tumors. IC play important roles in atherosclerosis and can either promote or reduce atherosclerosis progression (Hansson and Libby, 2006). It would be interesting to study whether we can use blood-based methylation profiles to predict the inflammation status of atherosclerotic plaques. We showed that the hypermethylated profile in atherosclerotic plaques described previously (Zaina et al., 2014), could be mainly attributed to increases in IC in the artery. This is of course not surprising as arteries and atherosclerotic plaques are a complex mixture of cell types and that atherosclerosis results in a dramatic remodeling of artery cell types, such as infiltration of IC and proliferation of smooth muscle cells. It is therefore questionable whether the methylation changes detected in atherosclerotic plaques are due to intrinsic methylation changes in specific cell types and whether these aberrant DNA methylation marks could be targets for cell type specific therapeutic interventions. We also need to point out that our reference methylome-based estimation of the cell type counts in plaques could not be validated with histologically determined cell type counts and that the tissue consist of much more complex cell types which were not included in the reference methylome. Furthermore, we used ENCODE cell lines as reference methylomes which may not be completely representative for the cells *in vivo*. However, previous studies already used cell lines to estimate cell type fractions, and a recent study used the same ENCODE cell lines to estimate cell type counts in aortic samples in relation to ascending aortic dissection and bicuspid aortic valve (Pan et al., 2017; Zheng et al., 2018). Overall, our immune-methylation profile may predict the immune status of solid tissues, and it should be further investigated whether IC changes detected in blood are also reflected in solid tissues. Again, more reference methylomes constructed by consortia such as BLUEPRINT and the International Human Epigenome Consortium (IHEC) may help detecting and accounting for rare cell subtypes in complex tissues.

Due to the lack of brain cell type reference methylomes, we were unable to estimate IC infiltration in AD brain tissues or the contribution of microglia. However, neuro-inflammation plays an important role in AD and there is evidence that systemic IC may infiltrate into the brain (Prinz and Priller, 2017). Whether our methylation profile correlates with neuro-inflammation or number of infiltrated IC should be further investigated. Interestingly, we found no correlation of methylation in AD cerebellum samples with our immune-DNA methylation signature, which is in accordance with studies showing that the cerebellum is less susceptible to AD neuropathological features like amyloid plaques and neuronal loss than cortex and hippocampus (Heneka et al., 2015).

Another limitation is that the whole blood and solid tissue samples are not obtained from the same individuals. We therefore don’t know whether a DNA methylation change in a person’s blood sample is also accompanied by a similar change in solid tissues. However, on average we observe that the consensus module obtained from blood samples is also prevalent in solid tissues.

In conclusion, inflammaging diseases, including atherosclerosis, AD, PD, and obesity, share a common DNA methylation profiles in whole blood samples representing a disease-associated immune component reflected by changes in blood IC counts and predictive for IC infiltration in disease tissues. In addition to epigenetic clock measurements, this immune-methylation signature may become a valuable blood-based biomarker to prevent chronic inflammatory disease development or monitor lifestyle intervention strategies which promote healthy aging.

## Data Availability Statement

Following publicly available datasets were analyzed in this study: GSE59685, GSE72778, GSE80970, GSE76105, GSE107143, GSE75434, GSE66500, GSE46394, GSE72774, GSE88824, GSE73103. 

## Author Contributions

KD and WVB contributed conception and design of the study. KD performed the data analyses. KD wrote the first draft of the manuscript. WB and KD wrote sections of the manuscript. Both authors contributed to manuscript revision, read and approved the submitted version.

## Funding

WVB and KD are supported by FWO grants G079614N and G059713N, grant Kom op tegen kanker (OZ7872), grant Stichting Alzheimer Onderzoek (OZ7953) and BOF NOI/DOCPRO/GOA grants (UA, FFB190077).

## Conflict of Interest

The authors declare that the research was conducted in the absence of any commercial or financial relationships that could be construed as a potential conflict of interest.
