# Supplementary material for: Characterization of Blood Surrogate Immune-Methylation Biomarkers for Immune Cell Infiltration in Chronic Inflammaging Disorders
Source: Front Genet. 2019 Nov 27;10:1229. doi: 10.3389/fgene.2019.01229 (PMC6890858; doi:10.3389/fgene.2019.01229)
Supplement: Supplementary file 1 [file DataSheet_1.docx]

Supplementary Material

# Supplementary Figures


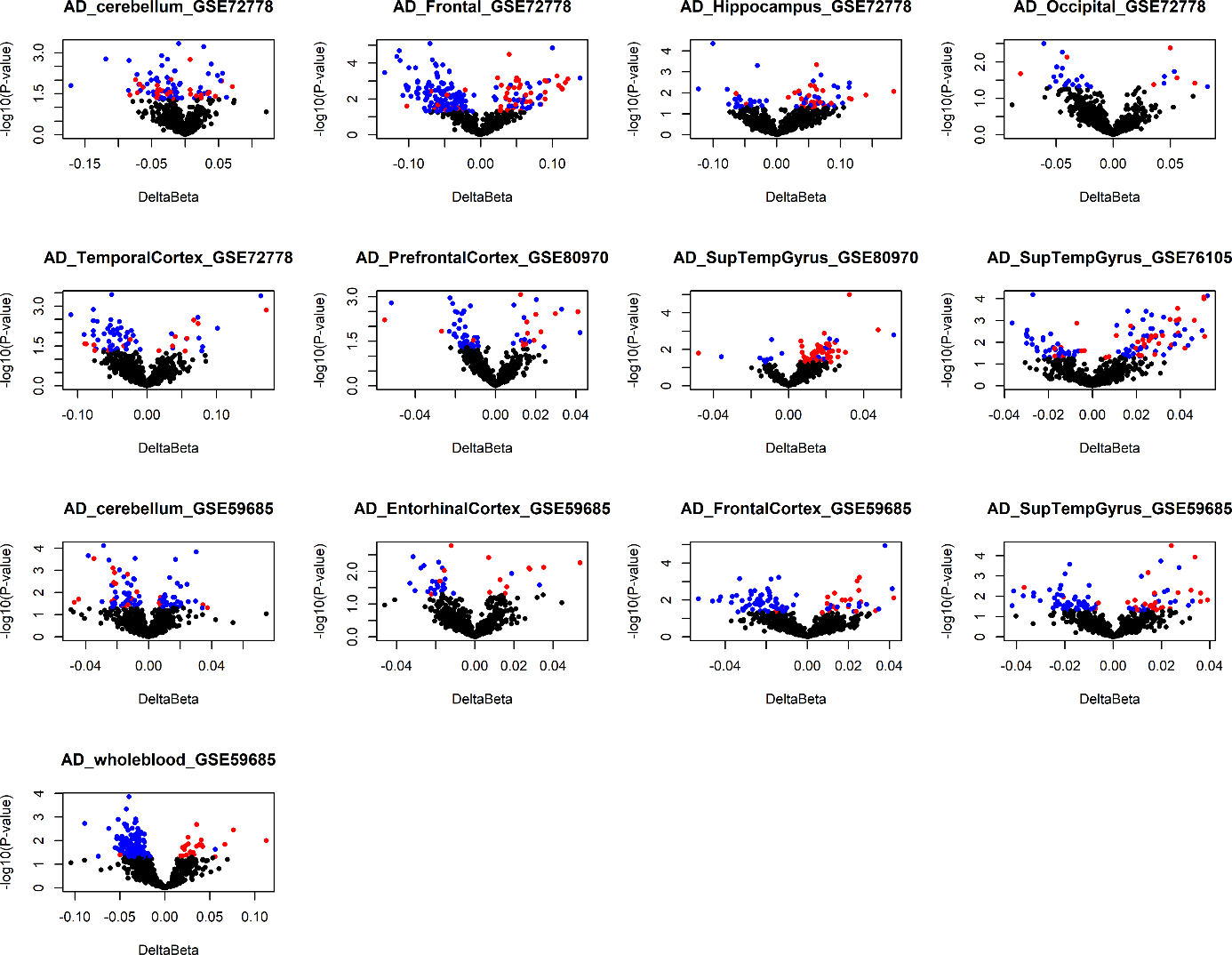


**Supplementary Figure 1:** DNA methylation similarity between atherosclerosis whole blood samples and Alzheimer’s disease brain tissues. Volcano plots showing the methylation differences and statistical significance values of the 712 top significant atherosclerosis DMPs (athero-DMPs) in different publicly available AD brain datasets comparing AD cases with healthy controls (see Table 1). Significant probes (p-value < 0.05) were colored blue when hypomethylated and red when hypermethylated in atherosclerosis.


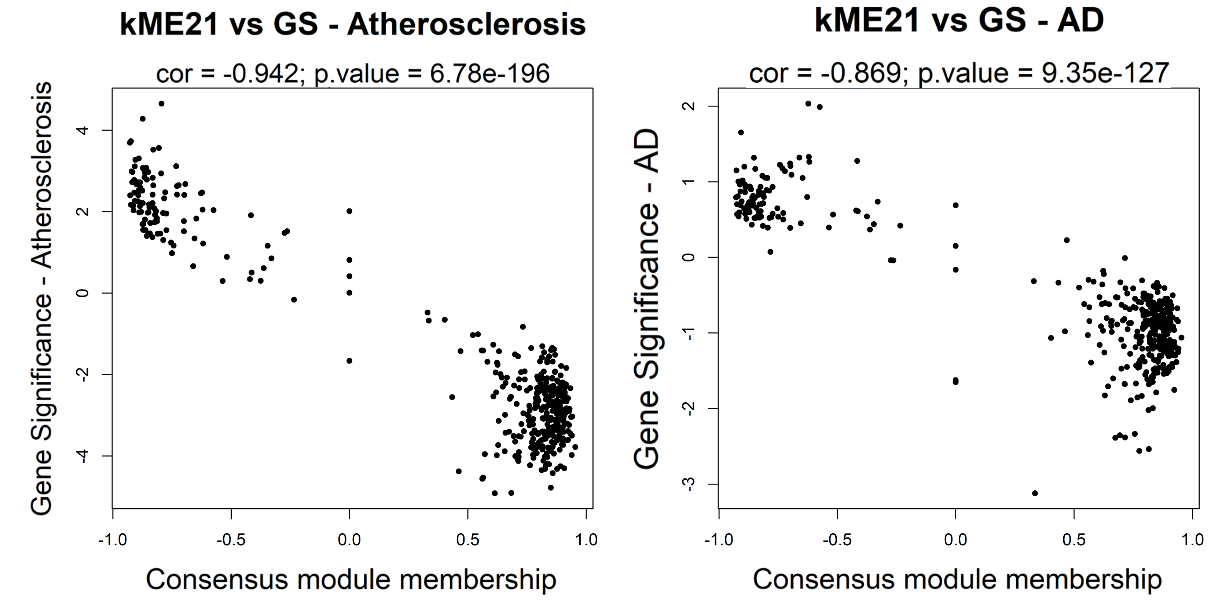


Supplementary Figure 2: Correlation between gene significance and module membership (kME21) of probes in the consensus module 21 in atherosclerosis (left) and AD (right). Pearson’s correlation coefficient and p-value is provided at the top of the plot.


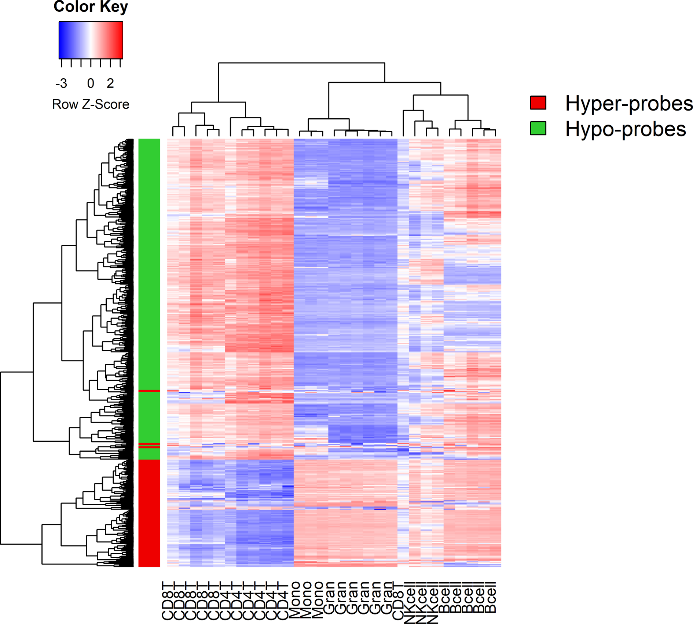


**Supplementary Figure 3:** DNA methylation values of the consensus module ME21 CpG probes across the major blood immune cell types. CpG probes which are hypo- and hypermethylated in atherosclerosis and AD are colored in green and red, respectively.


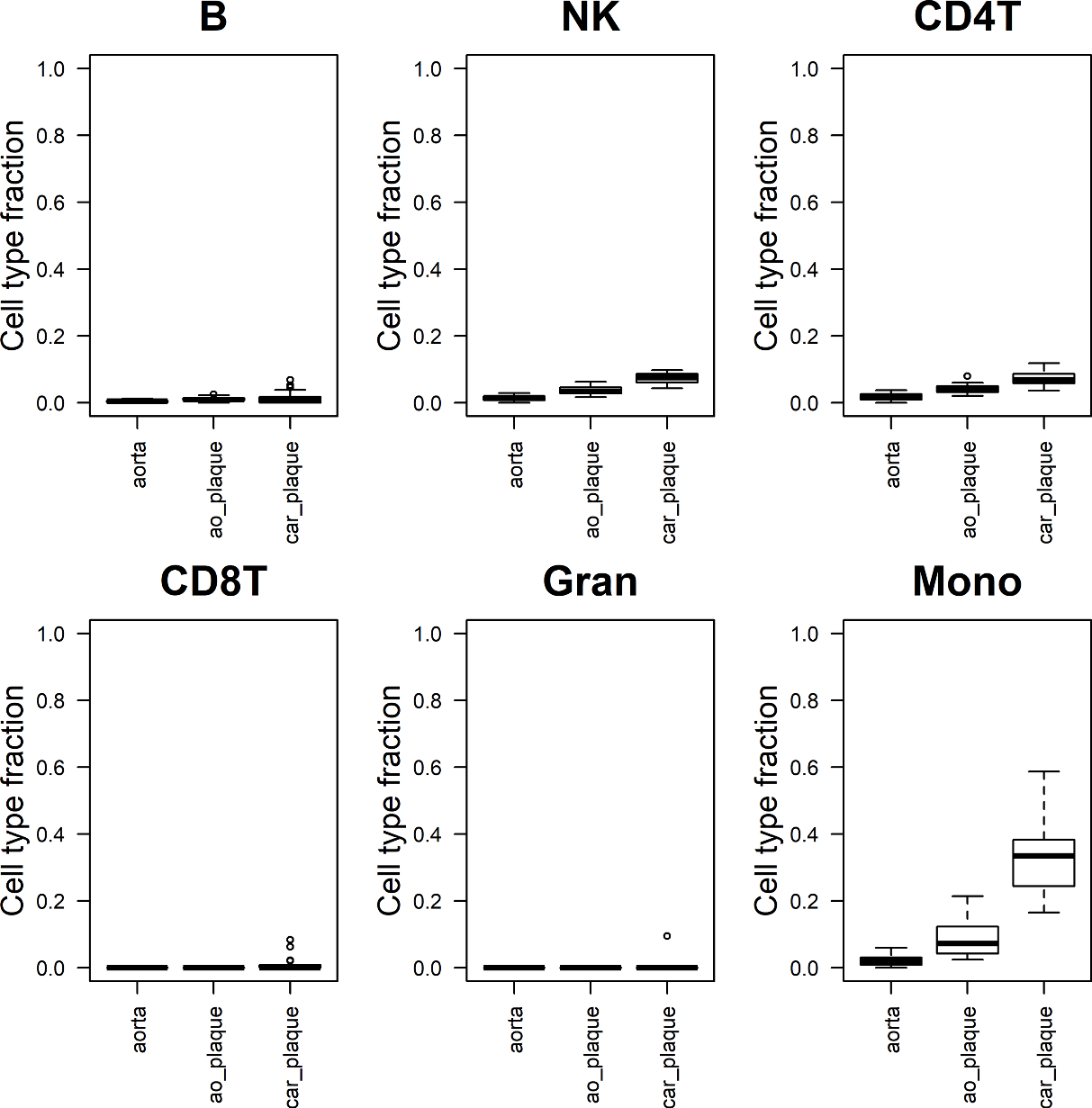


**Supplementary Figure 4:** Estimated immune cell fraction in healthy aorta, aorta atherosclerotic plaques (ao_plaque) and carotid plaques (car_plaque).


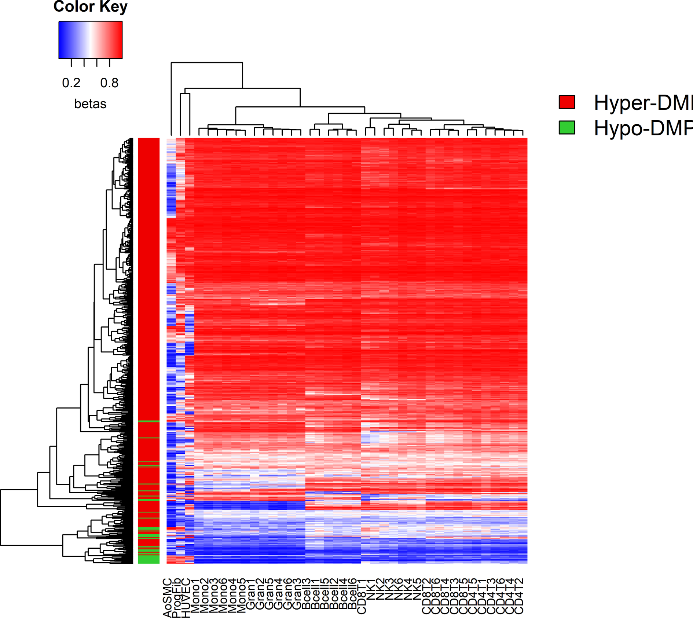


**Supplementary Figure 5:** DNA methylation levels of the top 500 most significant DMPs in aorta atherosclerotic plaques compared to healthy aorta tissues across different vascular and blood cell types (AoSMC: aorta smooth muscle cells, ProgFib: fibroblasts, HUVEC: endothelial cells). Hyper- and hypomethylated CpG probes in are colored in red and green respectively.


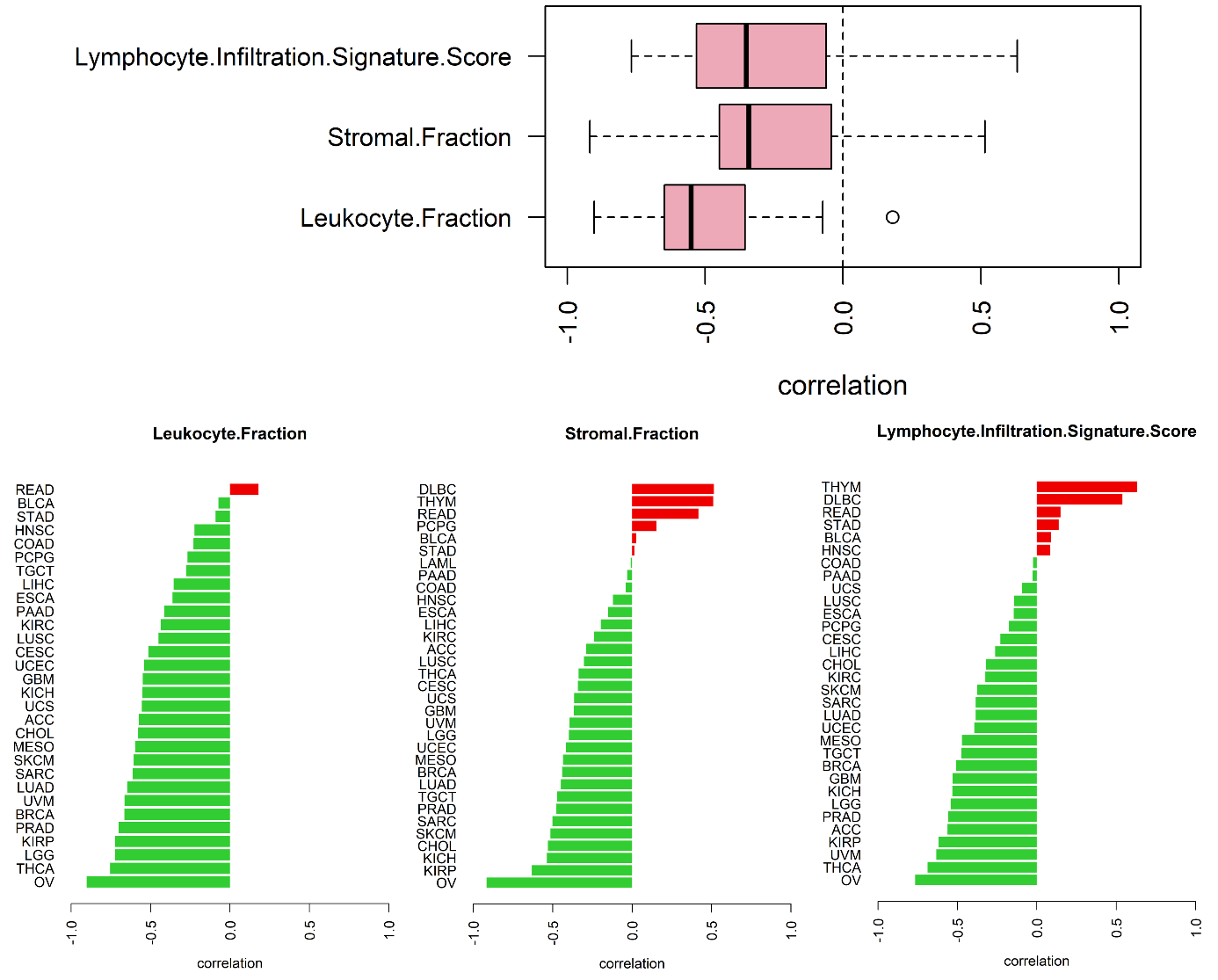


**Supplementary Figure 6:** TCGA immune cell infiltration. Pearson correlation between the consensus module eigengenes of TCGA cancers and measures of immune cell infiltration (leukocyte fraction, stromal fraction and lymphocyte infiltration signature score). The top panel represents the average correlation coefficients across all the TCGA cancers. The bottom panels show the correlation coefficients of each TCGA cancer separately for leukocyte fraction (left), stromal fraction (center) and lymphocyte infiltration (right).
